# Trends and factors associated with pregnancies among adolescent women in Nepal: Pooled analysis of Nepal Demographic and Health Surveys (2006, 2011 and 2016)

**DOI:** 10.1371/journal.pone.0202107

**Published:** 2018-08-09

**Authors:** Samikshya Poudel, Nawaraj Upadhaya, Resham Bahadur Khatri, Pramesh Raj Ghimire

**Affiliations:** 1 Ujyalo Nepal, Ratnanagar Municipality, Nepal; 2 Department of Research and Development, Health Works, Amsterdam, the Netherlands; 3 School of Public Health, The University of Queensland, Queensland, Australia; 4 School of Science and Health, Western Sydney University, New South Wales, Australia; The University of Warwick, UNITED KINGDOM

## Abstract

**Introduction:**

Adolescent pregnancy is a significant cost to mother, newborn, and their family and society. Despite the enormous health and social impact of adolescent pregnancy, there is a dearth of nationally representative studies on factors associated with adolescent pregnancies in Nepal. Therefore, this study aimed to examine trends and factors associated with adolescent pregnancies in Nepal, using pooled data of three nationally representative demographic surveys.

**Methods:**

Data for this study was derived from the recent three consecutive (2006, 2011 and 2016) Nepal Demographic and Health Surveys (NDHS). A total of 7,788 adolescent women aged 15–19 years included in the analysis. Trends and multivariable logistic regression analysis was performed to examine the factors associated with adolescent pregnancy.

**Results:**

Over the study period (2006–2016), the rate of adolescent pregnancy was 173 [95% Confidence Interval (CI): 159, 188] per 1000 women aged 15–19 years. Adolescent pregnancy was significantly higher among woman with middle household wealth index [adjusted Odds Ratio (aOR) 2.19, 95% confidence interval CI 1.65, 2.91] or poor household wealth index (aOR 2.37, 95% CI 1.76, 3.21). Similarly, Dalit (aOR 1.87, 95% CI 1.50, 2.34) or Madhesi (aOR 1.67, 95% CI 1.32, 2.11); and unemployed (aOR 1.28, 95% CI 1.09, 1.50) women had higher odds of adolescent pregnancies. In contrast, adolescent pregnancy was significantly lower among educated women (aOR 0.60, 95% CI 0.48, 0.74), and women with access to media exposure to public health issues (aOR 0.75, 95% CI 0.64, 0.88).

**Conclusions:**

Access to the media exposure on public health issues can be the effective efforts to reduce adolescent pregnancy. Women who have low maternal education, low wealth index, unemployed, and ethnic groups such as Dalits, and Madeshi needs to be targeted while designing and implementing policies and programs.

## Introduction

Globally, an estimated 16 million adolescent girls (15–19 years) give birth each year, and babies born to these adolescent mothers account for nearly 11% of births worldwide, with 95% occurring in developing countries such as Nepal[1, 2]. This high rate of adolescent pregnancies in developing countries can be explained by lack of knowledge and access to contraceptives, social norms (or even pressure) to marry early and bear child, lack of power to resist unwanted or coerced sex, and lack of safe abortion facilities. Most of these contextual factors give rise to unwanted adolescent pregnancy which has implications not only on the physical and mental health of the adolescent but also on socioeconomic prospect of her family and society. The health implications include unsafe abortion[1], pregnancy induced hypertension[3], eclampsia, puerperal endometritis, systemic infection, low birthweight babies, and preterm delivery[4]. The social implications on the other hand affect socioeconomic development of family and community because of early drop out from school, less job opportunities or diminish job prospects, and increased burden to families[5–7]. It is because of such health and social vulnerabilities that the mortality among adolescent women due to pregnancy and childbirth is almost doubled compared to women in their twenties[8]. Similarly, perinatal mortality is 50% higher among babies born to adolescent mothers than those aged 20–29 years[9].

South Asia is ranked second to Sub-Saharan Africa with the highest rate of adolescent pregnancy[10]. Practice of early marriage, poverty, and social expectation to have early child are considered key drivers of adolescent pregnancy in south Asia[6, 11]. Studies conducted in this region (including Nepal) revealed that lower socioeconomic status[12], rural residence[6], unintended pregnancy[13], illiteracy and working in agriculture[14] were associated with teenage pregnancy.

In Nepal, law and policies are in place to reduce adolescent pregnancy. For example, a law banning adolescent marriage (<20 years) became effective in 1963[15]. In 2000, Nepalese government adopted Adolescent Development and Health Strategy with the mandate of promoting health and socioeconomic status of adolescents through various measures including increasing age at marriage and reducing adolescent pregnancy[16]. Following this strategy, Nepalese women got legal access to have safe abortion in 2002, and the National Adolescent Sexual and Reproductive Health Program was initiated to roll out through its public health system in 2010[16]. Yet, adolescent marriage is still a common practice in Nepal[7]; and pregnancy and childbirth among Nepalese adolescents remain the second highest in South Asian region[17–21] which makes it even hard for Nepalese government to achieve health and socioeconomic related Sustainable Development Goal by the year 2030.

To reduce adolescent pregnancy, it is imperative to identify associated factors and incorporated into current national adolescent health strategy and program. In Nepal, studies around in-depth understanding of factors associated with adolescent pregnancy are limited. Previous studies that examined factors associated with adolescent pregnancy in Nepal were either hospital based or community based with small sample size, reducing the generalisability of findings to the whole of Nepalese population.

This study aimed to examine trends in adolescent pregnancy and investigate the factors associated with adolescent pregnancy in Nepal by pooling the most recent nationally representative household data of Nepal Demographic and Health Survey (NDHS) of the year 2006, 2011, and 2016. Evidence from this study can assist policy makers and program managers to formulate an integrated policy and programmatic response to meet the government strategic mandates geared towards reducing adolescent pregnancy and improving their health and socioeconomic prospect.

## Methods

### Data source

**Fig 1:**
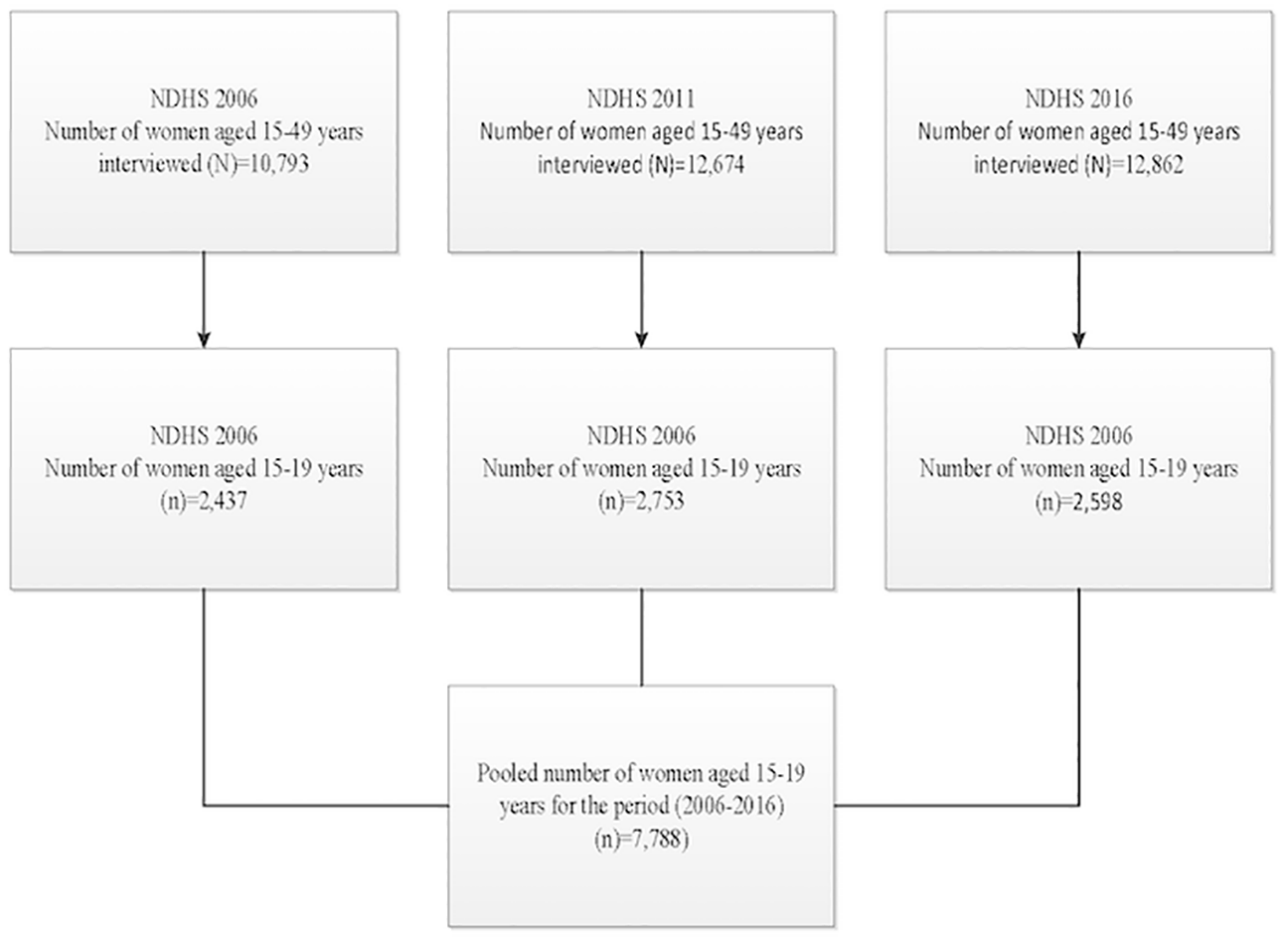
Flow chart for the selection of study population.

This study used pooled data of the 2006, 2011 and 2016 NDHS[17, 22, 23], conducted as a periodic estimate of the demographic and health indicators including information concerning various reproductive health issues such as pregnancy and childbirth using multi-stage cluster sampling design. The data was obtained from http://www.dhsprogram.com/data/available-datasets.cfm and the versions of the dataset used for this study were individual recode for women for the years 2006, 2011 and 2016. Data on pregnancy and childbirth history were collected from 36,329 women aged 15–49 years (10,793 in 2006[22], 12,674 in 2011[23], and 12,862 in 2016[17], that yielded an average response rate of 97%. For this study, we restricted our analyses to the 7,788 adolescent women aged 15–19 years (2,437 in 2006, 2,753 in 2011, and 2,598 in 2016) (Fig 1). Full details of sampling techniques used for obtaining the information has been published elsewhere[17, 22, 23].

### Variable definitions

In the reproduction section of women’s questionnaire of NDHS 2006, 2011, and 2016, women aged 15–49 years were asked to report past pregnancy outcomes as well as their current pregnancy status at the time of interview. Information on pregnancy outcomes and current pregnancy status were used to construct the outcome variable. The outcome variable of this study was adolescent pregnancy, defined as women aged 15–19 years who reported live birth or were pregnant with first child at the time of interview[17, 22, 23].

**Fig 2:**
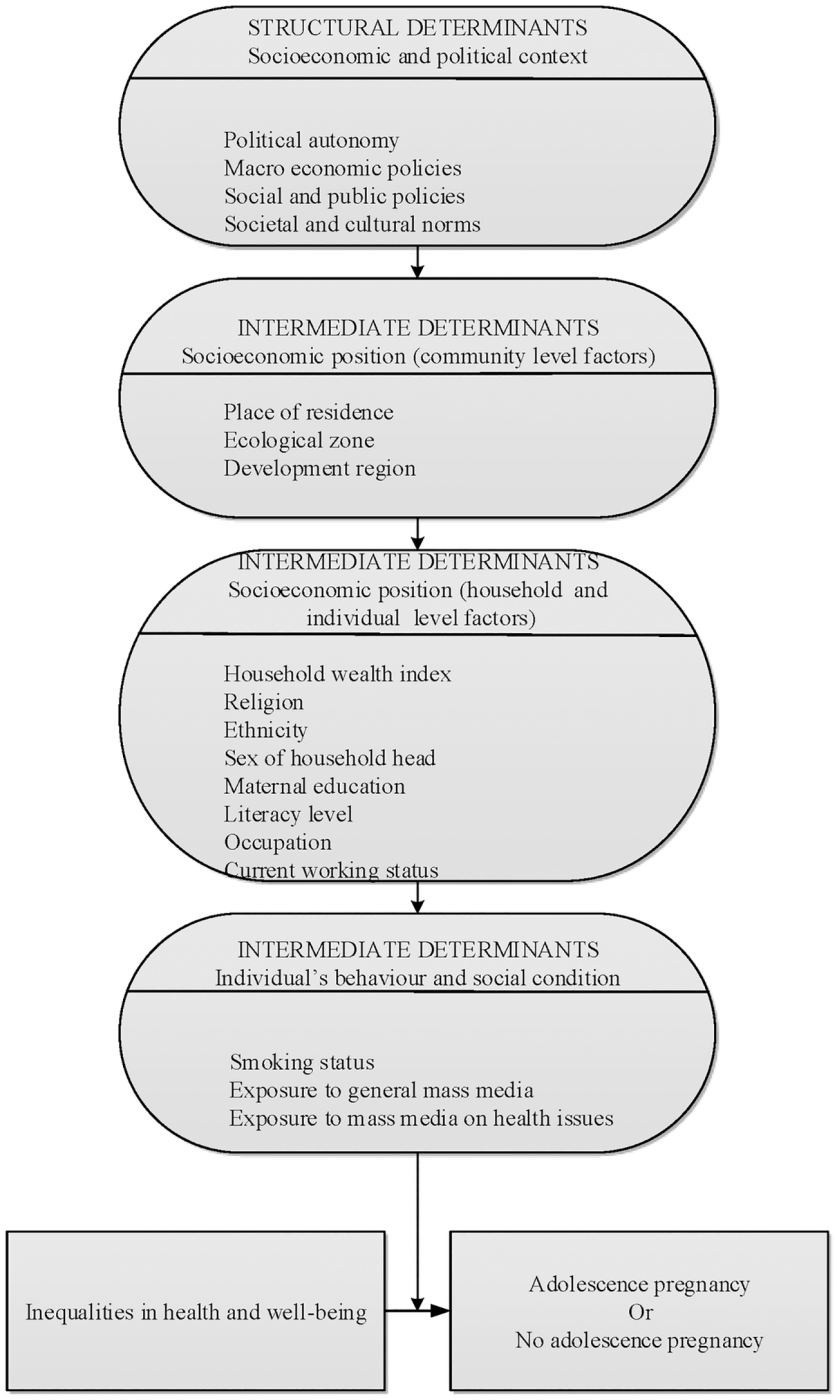
Modified conceptual framework for factors associated with adolescent pregnancy.

The selection of exploratory variables for this study was based on modified World Health Organization (WHO) conceptual framework[24] for social determinants of health inequalities and their impact on adolescent pregnancy, as well as information available in pooled NDHS dataset[17, 22, 23]. Past studies on adolescent pregnancy conducted in developing countries (including Nepal) also played role in the selection of potential exploratory variables[6, 11, 12, 25]. Based on the WHO conceptual framework, 14 exploratory variables were grouped into 3 categories: community level socioeconomic factors, household and individual level socioeconomic factors, and factors related to individual’s behaviour and social condition (Fig 2).

The community level socioeconomic factors included place of residence (rural and urban), ecological zone (terai, hill and mountain), and development region (western, eastern, central, mid-western and far-western).

The only household factor assessed was the household wealth index. Pooled wealth index factor scores[17, 22, 23] were used to construct wealth index variable (rich/middle/poor), consistent with previous studies[26, 27]. The individual level socioeconomic factors consisted of religion (Buddhist/Hindu/others including Muslim and Christian), sex of household head (male/female), women education (uneducated/educated), literacy level (can read part or whole of the sentence/cannot read), occupation (non-agriculture/agriculture/not working), current working status (working/not working), and ethnicity. Ethnicity was classified into four categories. Brahmin/Chettri is considered as advantaged ethnic group and belongs to the top of ethnic hierarchy whereas Dalit, Janajati, and Madhesi are relatively disadvantaged and socioeconomically marginalized groups[17, 22, 23, 28].

Factors related to individual’s behaviour and social condition examined were maternal smoking status (no/yes), exposure to general mass media (no access/have access), and exposure to mass media on health issues (no access/have access).

### Statistical analysis

Frequency tabulation was first performed to describe the characteristics of selected study variables. Adolescent pregnancy rate and 95% confidence interval was then calculated using ‘the number of adolescent pregnancy divided by number of adolescent women multiplied by 1000’.

Univariate logistic regression was conducted to examine unadjusted association between exploratory variables and study outcome. This was followed by multivariable logistic regression modelling to identify factors independently associated with adolescent pregnancy. For multivariable regression modelling, a three staged technique based on a conceptual framework described by Victoria et al was used[29]. First, all community level socioeconomic factors were entered into baseline multivariate model with manual backward elimination process to keep statistically significant factors (model 1). Second, all household level socioeconomic factors were entered into model 1 with manual backward elimination to keep statistically significant factors (model 2). In the third stage, factors related to individual’s behaviour and social condition were entered into model 2 with backward elimination to keep statistically significant factors in the final model (model 3). In each stage, the significance level was set at 0.05. All analyses were performed using STATA version 14.1 (Stata Corporation, College Station, TX, USA) with survey (SVY) commands for the adjustment of the cluster sampling survey design. Unadjusted and adjusted OR and their 95% CI were used to assess the factors associated with adolescent pregnancy. In the final model of multivariable logistic regression modelling, possible associated factors were examined for collinearity evidence.

### Ethics

Informed consent was obtained from all the study participants. The DHS project obtained ethical approvals from Nepal Health Research Council, Kathmandu, Nepal. The data sets used for this study were available to apply for on-line. The first author got permission from MEASURE DHS/ICF International to download and use the data for this study.

## Results

### Trends in adolescent pregnancy

**Fig 3:**
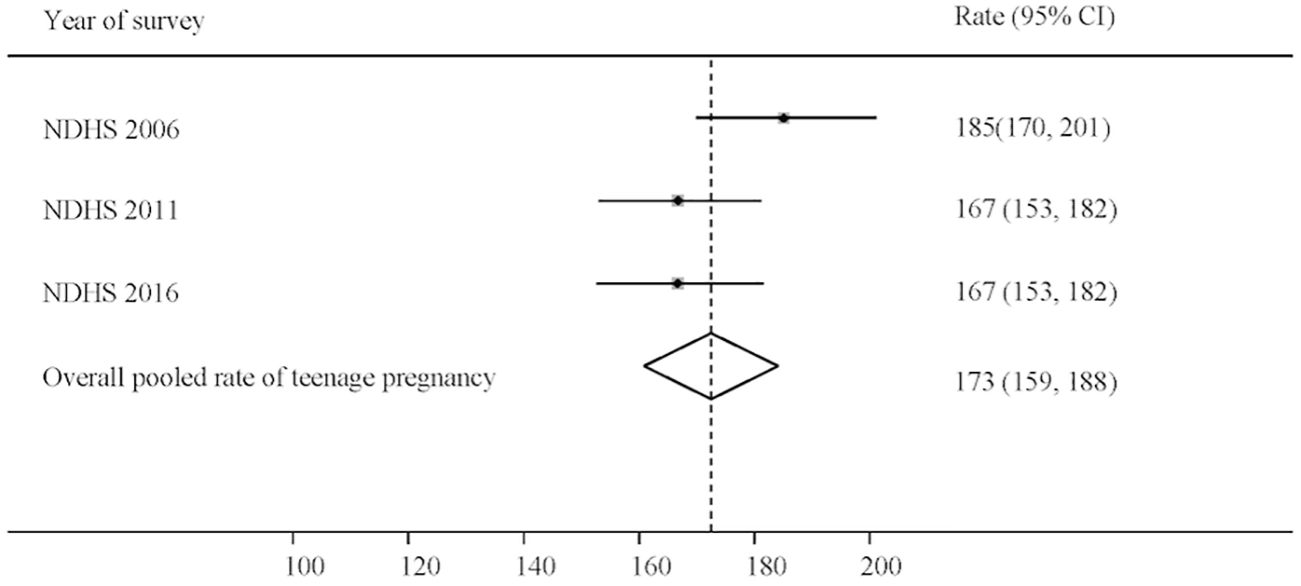
Trends in rate of pregnancy per 1000 women aged 15–19 years in Nepal (2006–2016). Note: NDHS: Nepal Demographic and Health Survey; CI: Confidence Interval.

While adolescent pregnancy rates have decreased slightly from 185 (95% CI: 170, 201) per 1000 in 2006 to 167 (95% CI: 153, 182) per 1000 in 2011, and remained constant until 2016, there has been no significant change in adolescent pregnancy rates in this period (Fig 3).

### Rates and factors associated with adolescent pregnancy

The analysis indicated that the rate of adolescent pregnancy varied between sub-population with socioeconomically better off having significantly lower pregnancy rate including those who were from the rich household, who belonged to higher ethnic group (Brahmin/Chettri), women with secondary and higher level of education, and those who had skilled or professional occupation (Table 1).

**Table 1:** Characteristics of study population as weighted counts, teenage pregnancy, and rates with 95% confidence interval in Nepal, NDHS 2006, 2011 and 2016 (N = 7788).

Study Variable	N	Adolescent pregnancy (n)	Rate (95% CI)
***Community level factor***			
Type of residence			
**Urban**	2320	303	131(123, 139)
**Rural**	5468	1040	190(181, 200)
Ecological zone			
**Terai**	4054	741	183(173, 192)
**Hill**	3207	507	158(149, 167)
**Mountain**	527	95	180(171, 190)
Development region			
**Eastern**	1779	309	174(164, 183)
**Central**	2555	459	180(170, 189)
**Western**	1543	246	159(151, 168)
**Mid-western**	999	196	196(186, 206)
**Far-western**	912	133	146(137, 154)
***Household and individual level factor***			
Household wealth index			
**Rich**	1607	142	88(82, 95)
**Middle**	3289	630	192(182, 201)
**Poor**	2892	571	197(188, 207)
Religion			
**Buddhist**	586	82	140(132, 148)
**Hindu**	6582	1138	173(164, 182)
**Others including Muslim and Christian**	620	123	198(188, 208)
Ethnicity			
**Brahmin/chettri**	2239	266	119(111, 126)
**Dalit**	1120	290	259(248, 270)
**Janajati**	2882	417	145(136, 153)
**Madhesi**	1547	370	239(228, 250)
Women education			
**Uneducated**	997	323	324(311, 337)
**Educated**	6791	1021	150(142, 159)
Women literacy level			
**Can read part or whole of the sentence**	6576	949	144(136, 153)
**Cannot read**	1212	394	325(312, 338)
Women occupation			
**Non-agriculture**	710	94	132(124, 140)
**Agriculture**	4197	713	170(161, 179)
**Not working**	2881	536	186(176, 196)
Working status			
**Working**	3772	598	159(150, 167)
**Not working**	4016	754	188(178, 197)
Sex of household head			
**Male**	5790	1038	179(170, 189)
**Female**	1998	305	153(144, 161)
***Individual Behaviour and social condition***			
Smoking status			
**No**	7691	1306	170(161, 179)
**Yes**	97	37	381(368, 395)
Access to general mass media			
**No access**	557	167	300(288, 312)
**Have access**	7231	1176	163(154, 172)
Access to mass media on public health issues			
**No access**	2664	639	240(229, 251)
**Have access**	5124	704	137(129, 146)

Univariate analysis revealed that type of residence (rural), household wealth index (middle or poor), ethnic minorities (dalit/janajati/madhesi), women education level (primary education or no education), women literacy level (illiterate), sex of household head (male), women who had access to general mass media, women who had access to mass media on public health issues, and those who reported of having smoking habit were significantly associated with increased adolescent pregnancy (Table 2).

**Table 2:** Unadjusted and adjusted odd ratios for factors associated with adolescent pregnancy in Nepal, NDHS 2006, 2011 and 2016.

Study Variable	Unadjusted Model	Model 1	Model 2	Model 3
	OR(95% CI)	OR(95% CI)	OR(95% CI)	OR(95% CI)
*Community level factor*				
Type of residence				
**Urban**	Reference	Reference		
**Rural**	1.57(1.28, 1.91)**	1.57(1.28, 1.91)**		
Ecological zone				
**Terai**	Reference			
**Hill**	0.84(0.69, 1.01)			
**Mountain**	0.98(0.77, 1.25)			
Development region				
**Eastern**	Reference			
**Central**	1.04(0.81, 1.35)			
**Western**	0.90(0.72, 1.14)			
**Mid-western**	1.16(0.93, 1.46)			
**Far-western**	0.81(0.58, 1.14)			
***Household and individual level factor***				
Household wealth index				
**Rich**	Reference		Reference	Reference
**Middle**	2.45(1.86, 3.23)**		2.23(1.68, 2.96)**	2.19(1.65, 2.91)**
**Poor**	2.55(1.92, 3.37)**		2.49(1.85, 3.36)**	2.37(1.76, 3.21)**
Religion				
**Buddhist**	Reference			
**Hindu**	1.28(0.92, 1.79)			
**Others including Muslim and Christian**	1.52(0.97, 2.36)			
Ethnicity				
**Brahmin/chettri**	Reference		Reference	Reference
**Dalit**	2.59(2.10, 3.18)**		1.98(1.60, 2.47)**	1.87(1.50, 2.34)**
**Janajati**	1.25(1.02, 1.55)*		1.15(0.93, 1.43)	1.15(0.92, 1.42)
**Madhesi**	2.33(1.85, 2.92)**		1.82(1.45, 2.30)**	1.67(1.32, 2.11)**
Women education				
**Uneducated**	Reference		Reference	Reference
**Educated**	0.37(2.17, 3.07)**		0.53(0.43, 0.66)**	0.60(0.48, 0.74)**
Women literacy level				
**Can read part or whole of the sentence**	Reference			
**Cannot read**	2.86(2.43, 3.38)**			
Women occupation				
**Non-agriculture**	Reference			
**Agriculture**	1.35(0.92, 1.98)			
**Not working**	1.50(0.98, 2.29)			
Working status				
**Working**	Reference		Reference	Reference
**Not working**	1.21(1.02, 1.43)		1.28(1.09, 1.50)*	1.28(1.09, 1.60)*
Sex of household head				
**Male**	Reference			
**Female**	0.83(0.69, 0.99)*			
***Individual behaviour and social condition***				
Smoking status				
**No**	Reference			Reference
**Yes**	3.02(1.90, 4.84)**			2.70(1.64, 4.45)**
Access to general mass media				
**No access**	Reference			
**Have access**	0.45(0.36, 0.58)**			
Access to mass media on public health issues				
**No access**	Reference			Reference
**Have access**	0.51(0.43, 0.59)**			0.75(0.64, 0.88)**

**: p<0.001;

*: p<0.05

Multivariable analysis found that the odds of adolescent pregnancy were significantly higher among women who came from middle or poor household, who belonged to ethnic groups of dalit or madhsi, who had primary or no education, who had access to mass media on public health issues, and those who reported of having smoking habit (Table 2).

In the final model, when we replaced household wealth index by type of residence, the result showed that adolescent women living in rural area were significantly more likely to become pregnant aOR: 1.33 (95% CI: 1.08, 1.63, P-value: 0.007) compared to those living in urban area. Similarly, when we replaced women education by women literacy level, the result indicated that illiterate adolescents were significantly more likely to become pregnant aOR: 1.77 (95% CI: 1.45, 2.16, P-value: <0.001) compared to their literate counterparts.

## Discussion

Despite a major contributor to adverse maternal and child health outcomes, the rate of adolescent pregnancy remains very high in Nepal. We found that adolescent pregnancy differed significantly on the basis of different forms of social marginalization such as geography, ethnicity, employment status, and household economic background. In the community level, rural residence was found to be associated with higher adolescent pregnancy. The household and individual level factors associated with increased adolescent pregnancy were women who belonged to lower ethnic stratum (dalit /madhesi), who did not have employment, and those who were from middle or poor household. The behaviour and social condition factors found to be associated with adolescent pregnancy were women who reported of having smoking habit and those who had access to mass media on public health issues.

Our study revealed that women who resided in rural areas were significantly associated with higher adolescent pregnancy compared to those who resided in urban areas. This finding is consistent with previous studies conducted in Bangladesh[6]; and similar argument indicated by Sayem et al that the higher odds of adolescent pregnancy in rural Nepal may be attributed to the fact that marriage among rural women takes place earlier than those who live in urban[17]. The practice of early marriage among rural women may be driven by firmly established traditional beliefs that are widely practiced in many parts of rural Nepal. For instance, in rural Nepal, the belief of early girl marriage for social acceptance is still common[30]; and in many occasions, the decision of early marriage is taken by their parents[30]. Community education program around negative health impact of early marriage, targeted towards rural parents and those who are involved in marriage decision, can help increase marital age and reduce adolescent pregnancy because 70% of the Nepalese adolescents inhabit in rural Nepal.

Household economic status significantly influenced the odds of adolescent pregnancy. Consistent with systematic reviews[12, 31, 32], we found that adolescents who belonged to middle or poor household had higher odds of being pregnant compared to those who belonged to rich household. This is not only the case for developing countries; the lower economic status has been consistently reported as a significant factor of teenage pregnancy in developed countries as well[33]. The higher odds of adolescent pregnancy among women of poor household in Nepal may be aggravated due to economic burden that are often placed on women while they grow older. For example, the dowry system is still prevalent in Nepal. As part of dowry system, bride family are required to offer more dowry when daughter grows older[34]; which should put women under a lot of pressure for early marriage. While this may be true that employed girls can economically support the poor family and can remain in the family for longer age, our study also found significantly lower pregnancy among adolescents who reported having employment.

In contrast to protective effect of education against adolescent pregnancy in the current study, a cross-sectional study conducted in Bangladesh[6] has shown no significant protective effect of education against adolescent pregnancy. This result from Bangladeshi study could have been attributed to methodological limitation such as small sample size and assessment of older women (20–29) that constitute almost 74% of the study sample. Our finding of higher risk of pregnancy among uneducated or illiterate women is consistent with studies conducted in developing countries including Nepal[25, 35, 36]. It is possible that educated or literate women are more empowered and better informed about their fundamental and legal rights that are essential to make logical decision about healthy life including those that are essential to deny early marriage and early pregnancy.

Ethnicity impacts adolescent pregnancy in Nepal. Our study revealed that women from the lower ethnic background (Dalit or Madhesi) were significantly more likely to report pregnancy during adolescence compared to those of higher ethnic background (Brahmin and Chettri). This finding is similar to those reported in previous studies conducted in low-and middle-income countries[30, 31, 37]. The association of teenage pregnancy with ethnic minorities in our study may be due more to its association with education and socioeconomic status than with a direct effect of ethnic class. For example, of 16% illiterate women who reported teenage pregnancy, only 1% were from Brahmin/Chettri ethnic background compared to 5% from Dalit, 3% from Janajati, and 7% from Madhesi ethnic backgrounds[17, 22, 23].

Our findings showed that women who reported having smoking habit were significantly more likely to become pregnant during teenage period compared to women without smoking habit. Studies that have examined the association between smoking habit and teenage pregnancy are scarce in developing countries. However, our finding of higher teenage pregnancy among smokers is consistent with a study conducted in rural England[38]. Smoking during teenage period is considered a risk-taking behaviour; and our finding of higher teenage pregnancy among women who reported smoking habit may be the reflection of such risk taking behaviour.

The present study revealed that women who had access to mass media on public health issues were protective against adolescent pregnancy, and this finding is consistent with a cross-sectional study conducted in Bangladesh[6]. It is possible that women who have access to public health mass media are more likely to acquire knowledge about important public health issues including those of family planning, as well as the risk of teenage pregnancy and childbirth. The translation of such knowledge into practice may have further attributed for such protective effect on adolescent pregnancy.

The study findings call for policy level actions such as health promotional programs around negative health impacts of early marriage and pregnancy targeted not only to adolescent girls but also to the elders who make the marriage decision to their children/relatives. Such programs need to be scaled up in rural areas with special focus for the poor and marginalised communities, Dalit and Madhesi in particular. As access to public health messages was protective factor for adolescent pregnancy, the Ministry of Health and Population needs to promote and implement the Information, Education, and Communication (IEC) related interventions through school curricula, community sensitization events and mass media.

This study has used pooled NDHS data for the period (2006–2016) with bigger sample size and increased statistical power. Additionally, the NDHS data used for the present study are nationally representative with average response rate of 97% that the selection bias is unlikely to affect the study findings. Finally, the same core questionnaires were used to collect data for the year 2006, 2011 and 2016; and the surveys were conducted by same organizations that increase the consistency and the accuracy of data[17, 22, 23]. However, the following limitations should be taken into account while interpreting the study findings. Firstly, the information provided by the respondents was based on self-report that may be subject to recall bias. Secondly, the analyses were based on cross-sectional data, and hence the establishment of clear temporal relation between study outcome and confounding factors cannot be determined.

## Conclusions

This study highlights the trends in adolescent pregnancy over a 15 year period as well as the role of socioeconomic condition in which women live with, and their association with adolescent pregnancy. This study found non-significant reduction of teenage pregnancy over a 15 year period. Interventions of improving health knowledge can help reduce adolescent pregnancy. Due to multifactorial nature of its determinants, these interventions should be targeted mainly for adolescents with no education, who belong to poor household, who live in rural areas, and those who are of dalit and madhesi ethnic background.
